# Contralateral C7 nerve transfer - Our experiences over past 25 years

**DOI:** 10.1186/1749-7221-6-10

**Published:** 2011-11-23

**Authors:** Cheng-Gang Zhang, Yu-Dong Gu

**Affiliations:** 1 Department of Hand Surgery, Huashan Hospital, Fudan University, Shanghai, People's Republic of China; 2 Key Laboratory of Hand Reconstruction, Ministry of Health, Shanghai, People's Republic of China; 3 Shanghai Key Laboratory of Peripheral Nerve and Microsurgery, Shanghai, People's Republic of China

## Abstract

Contralateral C7 nerve transfer has been used in treating brachial plexus avulsion injury since 1986. During the past two and half decades, much has been achieved, yet more needs to be explored. In this review article, the indications, technical details, outcome and pitfalls of this technique are summarized.

## Introduction

It has been 25 years since the world's first case of contralateral C7 nerve transfer finished in our clinic in August 1986 [1]. We now summarize our experiences regarding this technique.

Brachial plexus avulsion injury represents one of the most devastating injuries of the upper extremity. Nerve transfer is the most frequently used method in restoring limb function. So far, various techniques have been used, intraplexus or extraplexus, including accessory nerve transfer, intercostal nerve transfer, phrenic nerve transfer etc. However, with the fast development of high-velocity traffic, there have been increasing high-energy accidents over the recent years which resulted in more extensive trauma. In these cases, even fewer donor nerves could be used in neurotization. This prompts us to seek more donor sources for brachial plexus reconstruction.

## Clinical case

In June 1986, a 28-year old man sustained hemopneumothorax due to 3rd-6th costal fractures and brachial plexus injury in the left side during a motor cycle accident. He was referred to our clinic 2 months after initial trauma due to there has been no spontaneous recovery of the upper limb function. Upon physical examination, a positive Claude Bernard-Horner's sign was found. The function of the shoulder, elbow, wrist and hand was completely lost. Plain chest X-ray film also showed elevation of the left diaphragm. Electromyogram detected no SEP from C5-T1 nerve roots and NAP could be recorded. The diagnosis was preganglionic injury of the C5-T1 nerve roots, ie., total root avulsion injury of the brachial plexus. The EMG exam also suggested concomitant complete palsy of the accessory nerve and phrenic nerve. Based on our observation of over 1000 cases of brachial plexus injuries, no patient suffered functional loss from single C7 root injury, therefore we postulated that C7from the healthy limb may be sacrificed and used as a donor nerve to reconstruct the injured plexus. During surgical exploration of the affected plexus, C5-T1 nerve roots were found avulsed. Due to extensive scarring in the neck/shoulder region, the accessory nerve and motor branches of the cervical plexus could not be used. The phrenic nerve was found to be buried in the scarry tissue on the surface of the fibrosed anterior scalenus muscle and a neurolysis was performed.Upon electrical stimulation a strong contraction of the diaphragm could be induced which indicated the viability of phrenic nerve. It was then divided with its proximal stump transferred directly to the anterior division of the upper trunk (fascicles destined to the musculocutaneous nerve). Since there were no further donor nerves to be used in the injured side, the contralateral C7 nerve was decided to be used and therefore exposed and divided. In the injured side, the ulnar nerve was freed from the wrist level to upper arm and then transposed and tunnelled subcutaneously to the incision in the healthy side to be sutured with the contralateral C7 nerve. The ulnar vessels were dissected along with the ulnar nerve and the anastomosis was performed: ulnar artery with the transverse cervical artery and ulnar vein with a tributary of the external jugular vein.

On first postoperative day, sensory deficits were found in the index finger and middle finger and motor function of the healthy upper limb was normal. The muscle strength of latissimus dorsi, triceps brachii, extensor digitorum communis, flexor carpi ulnaris and extensor carpi radialis was all above M4. The grip strength was 36 kg (40 kg before operation) and the pinch strength was 4 kg (same as before operation). The patient did not manifest any signs of respiratory disorder. The paraesthesia in the healthy hand disappeared 2 weeks later and the grip strength returned to 40 kg and all joint function of the healthy side was normal.

In October 1987, 14 months after the first stage, percussion along the ulnar nerve from the healthy side toward the injured side showed Tinel's sign positive at the midpoint of the upper arm indicating the regeneration reached the site, which corresponded with the regenerating rate of approximately 1 mm per day, (38 cm over 420 days). The muscle strength of the biceps recovered to M3. Therefore the regenerated ulnar nerve was divided in the upper arm and transferred to the median nerve. In February 1989, 30 months after first stage, the muscle strength of the flexor carpi radialis recovered to M3, the flexor digitorum superficialis of 2nd - 5th fingers was M2, and sensation recovered to S2 in the radial 3 fingers. The muscle strength of biceps was M4 and the patient could freely flex the elbow without initiated by respiration. In August 1990, 4 years after first stage, the muscle strength of flexor carpi radialis, palmaris longus and flexor digitorum superficialis was M4, flexor digitorum profundus and flexor pollicis longus was M3 and sensation in the radial 3 fingers recovered to S3.

Therefore we conclude the indications for contralateral C7 transfer as follows:

1, No available neurotizers in the affected side;

2, Used as one of the neurotizers in multiple neurotization in total root avulsion injury;

3, When one of the multiple neurotizations fails for any type of avulsion injury

## Technical aspects

Under general anesthesia, the patient is put in supine position.

Dividing contralateral C7 nerve

The brachial plexus of the healthy side is explored through a transverse incision parellel to the clavicle (about 2 cm above the clavicle) starting from the posterior margin of the sternocleidomastoid muscle. The small branches of the external jugular vein can be ligated and the omohyoid muscle is divided and retracted to the side. The transverse cervical vessels are ligated and the C5-T1 nerve roots are exposed. Before dividing C7, a little amount of 2% lidocaine is injected epineurially to protect the proximal neurons. The C7 nerve can be severed at the common trunk level or its posterior division or anterior division level depending on the diameter of the ulnar nerve graft and the recipient nerve to be reconstructed.

## Harvesting ulnar nerve graft

In the injured side, the ulnar nerve (the main trunk and the dorsal cutaneous branch) is cut at the wrist level and freed proximally to the upper arm. Attention needs to be paid to protect the superior ulnar collateral vessel for blood supply to the upper segment of the ulnar nerve graft. Then a cross-chest subcutaneous tunnel is made to bring the ulnar nerve to the divided contralateral C7 for a tension-free nerve suture.

The patient is required to wear a head-shoulder spica for 4 weeks after operation to prevent rupture of the nerve suture.

## Second stage transfer of ulnar nerve

When nerve regeneration from contralateral C7 has reached axilla of the affected side as judged by clinical and electrophysiological studies, usually about 10 months, the ulnar nerve is divided in the upper arm and transferred to the recipient nerve [2,3].

## Results from different authors

In our adult series of contralateral C7 transfer followed up for over 2 years, the overall motor recovery rate (> = M3) was 50-80% depending on different recipient nerves and the sensory recovery rate (> = S3) was above 60%. Table 1 shows the demographic data of the cases and Table 2 shows the functional recovery based on various recipient nerves. In our separate series on infants and children, noteworthy function was achieved in 10 of 12 patients and sensory function was gained in all patients (age 6-93 months, average 17 months, followed up for a mean of 42 months) [4].

**Table 1:** Demographic data of the cases

Male	47
Female	15
Left side	36
Right side	26
Age	16-50 years
	(Average: 27.8 years)
Causes	
Traffic accident	47
Machine traction injury	8
Fall from height	4
Heavy object falling on the shoulder	3

**Table 2:** Functional recovery of contralateral C7 nerve transfer

		Motor Recovery				Sensory Recovery			
Target nerve	**No**.	M4	M3	M2-1	M0	S4	S3	S2-1	S0
Musculocutaneous N	14	5	6	2	1	2	8	3	1
Median N	36	8	12	9	7	4	19	8	5
Radial N	10	3	2	3	2	0	7	1	2
Thoracodorsal N	2	1	0	0	1	0	0	0	0

Terzis recently reported the fair (M2+~M3), good (M3+~M4-) and excellent (M4+~M5-) rates of 56 cases were 74% for biceps; 57% for triceps; 50% for deltoid; 62% for wrist and finger flexors and 50% for wrist and finger extensors, respectively [5].

But in a report of 96 cases by Waikakul, only 52% of patients had > = M3 recovery after contralateral C7 transfer to musculocutaneous nerve, and 20% recovery for the extensor of wrist/finger and 29% recovery for finger flexor [6]. Sammer et al reported results from 2 groups of hemi-contralateral C7 transfer, that no patient developed useful function after median nerve repair and only 23% > = M3 recovery after suprascapular or axillary nerve repair [7]. The most optimistic result was reported by Hierner that 100% M3 biceps recovery was achieved in 6 patients while for median nerve the recovery rate was 25% [8].

Various results obtained from different authors might be contributed to a few factors. Apart from patient age and surgical delay, we consider the following 2 factors are of paramount importance to the good surgical outcome.

1, Fascicle selection of contralateral C7: anterior division, posterior division or whole C7?

In most of our patients, the ulnar nerve is used as nerve graft. And in the majority of the cases, the diameter of C7 exceeds that of the ulnar nerve. Therefore, sometimes partial C7 was used for neurotization. In a previous study, it has been demonstrated that posterior division of C7 contains more motor fibers than anterior division [9]. Therefore if the aim of the transfer is to restore motor function eg wrist/finger flexion, the posterior division should be used and if the sensory function is desired, eg. to restore protective sensibility of hand, the anterior division should be used. This may also explain the low motor recovery rate in Waikakul's series since only anterior division was used to neurotise median nerve while the sensory recovery was good (83%) in his group. Sammer et al also used hemi-C7 to repair median nerve (0%) and suprascapular/axillary nerve (23%). Therefore we prefer using whole C7 for neurotization to fully utilise the large source C7 can provide, which was also the way Terzis did in her series [4].

2, Vascularization of the nerve graft

In adults, the distance between contralateral C7 and recipient nerve in the injured arm is over 30 cm. In such case, the blood supply of the long nerve graft is essential to maintain the regenerative potential of C7. Therefore, we strongly advocate performing contralateral C7 transfer in 2 stages. In the first stage, the distal end of ulnar nerve is transposed and connected with contralateral C7 to allow nerve regeneration, while the proximal part of ulnar nerve is intact to preserve blood supply of the ulnar nerve graft. This might be another reason for the low recovery rate in Waikakul's and Sammer's series, when C7 was immediately transferred to median nerve and/or axillary nerve. We have also compared different patterns of bridging the C7 to the recipient nerve: free sural nerve graft, sural nerve graft vascularised through saphenous vein, pedicled ulnar nerve based on superior ulnar collateral artery and ulnar nerve graft with anastomosis of ulnar vessels. The results appear to be better in the patients with better blood supply of the nerve graft [1,10,11]. It has also been proved that when the diameter of superior ulnar collateral artery was over 0.5 mm, it could provide sufficient blood supply to full-length ulnar nerve (length/width ratio about 45:1) [12], Therefore we prefer using this vessel instead of performing ulnar artery and vein anastomosis [13].

## Safety in dividing contralateral C7

C7 forms middle trunk and no single muscle in the upper limb is innervated solely by C7. Therefore, dividing C7 will cause no permanent loss in sensory and motor function. Usually, the patients will undergo numbness in the fingers in the first 3 months after operation. The most affected fingers are index finger (74%), middle finger (58%) and thumb (38%) [14]. There will be temporary decrease in the grip strength but the pinch strength is not affected [15]. It is worth pointing out that the C7 transection site should not be too distal (should never go infraclavicularly) and otherwise the fibers from upper and lower trunk may be injured and permanent motor and sensory deficits will be caused [12].

## Multiple use of contralateral C7 transfer

One single C7 nerve carries vast nerve fibers that exceed total number from the frequently used donor nerves such as accessory nerve, phrenic nerve and intercostal nerve. Therefore, it is reasonable to use it to neurotise more than 1 recipient nerve [4]. In a preliminary clinical study on double use of contralateral C7 transfer, acceptable recovery was achieved in C7 to musculocutaneous nerve and median nerve, while the result was poor in C7 to median and radial nerve [16]. This is probably due to the brain plasticity transformation is more difficult for 2 nerves of antagonistic functions, eg. finger flexion and extension. Therefore the double use of contralateral C7 transfer should only be cautiously used in well selected cases and two recipient nerves are better of synergistic functions, eg. elbow flexion and finger flexion.

Cross-chest subcutaneous route vs prespinal route contralateral C7 transfer

Mcguiness reported one case of prespinal route in contralateral C7 transfer in 2002 [17] and other author also reported their experience of this technique more recently [18]. The advantage of this route is it saves the regeneration distance of nerve graft and therefore early functional recovery is expected. However, it puts patients under risk in making the retropharyngeal space to pass the graft. The benefits from this route, together with the safety of this technique (eg, bleeding, phrenic nerve or esophagus injury) are to be evaluated on these early cases after longer observation period.

## Brain plasticity in contralateral C7 nerve transfer

After contralateral C7 nerve transfer, the patient is encouraged to perform more exercises of the healthy limb especially elbow extension and shoulder adduction thus to stimulate regeneration from contralateral C7 toward the injured side along the nerve graft. In the early stage of functional recovery, all patients experience problem with involuntary movement of the injured arm-the motion has to be initiated by the movement of the healthy arm. In majority of patients this phenomenon lasts for 5 years and usually independent movement can be obtained when the muscle strength recovered to M3 [2]. Brain plasticity plays an important role in the transformation process. With the advanced brain imaging technology such as fMRI and PET scan, it is now possible to look into the changes in the brain and contralateral C7 transfer opens a unique venue to study the connections between 2 hemispheres.

## Competing interests

The authors declare that they have no competing interests.

## Authors' contributions

CGZ carried out the followup study and collection of data. YDG conceived of the study and participated in its design and coordination. All authors have read and approved the final manuscript.
